# User-Centered System Design for Communicating Clinical Laboratory Test Results: Design and Evaluation Study

**DOI:** 10.2196/26017

**Published:** 2021-11-25

**Authors:** Zhan Zhang, Lukas Kmoth, Xiao Luo, Zhe He

**Affiliations:** 1 School of Computer Science and Information Systems Pace University New York, NY United States; 2 School of Engineering & Technology Indiana University–Purdue University Indianapolis Indianapolis, IN United States; 3 School of Information Florida State University Tallahasse, FL United States

**Keywords:** clinical laboratory results, patient-centered care, patient portal, health communication

## Abstract

**Background:**

Personal clinical data, such as laboratory test results, are increasingly being made available to patients via patient portals. However, laboratory test results are presented in a way that is difficult for patients to interpret and use. Furthermore, the indications of laboratory test results may vary among patients with different characteristics and from different medical contexts. To date, little is known about how to design patient-centered technology to facilitate the interpretation of laboratory test results.

**Objective:**

The aim of this study is to explore design considerations for supporting patient-centered communication and comprehension of laboratory test results, as well as discussions between patients and health care providers.

**Methods:**

We conducted a user-centered, multicomponent design research consisting of user studies, an iterative prototype design, and pilot user evaluations, to explore design concepts and considerations that are useful for supporting patients in not only viewing but also interpreting and acting upon laboratory test results.

**Results:**

The user study results informed the iterative design of a system prototype, which had several interactive features: using graphical representations and clear takeaway messages to convey the concerning nature of the results; enabling users to annotate laboratory test reports; clarifying medical jargon using nontechnical verbiage and allowing users to interact with the medical terms (eg, saving, favoriting, or sorting); and providing pertinent and reliable information to help patients comprehend test results within their medical context. The results of a pilot user evaluation with 8 patients showed that the new patient-facing system was perceived as useful in not only presenting laboratory test results to patients in a meaningful way but also facilitating in situ patient-provider interactions.

**Conclusions:**

We draw on our findings to discuss design implications for supporting patient-centered communication of laboratory test results and how to make technology support informative, trustworthy, and empathetic.

## Introduction

### Motivation

Health care organizations are increasing direct access of patients to their clinical data via patient portals [1-3]. For example, patients can check their laboratory test results, an important type of medical record data, on the portals outside of the clinical environment (eg, at home). It has been shown that providing patients with access to such data can lead to better patient-centered medical care [1,4-6] and enhanced patient-provider relationships [7,8]. Despite these potential benefits, the literature points out that merely providing access to laboratory test results is insufficient to improve patient engagement in their care because many patients are not able to make sense of the data and, as such, their use of laboratory test results is significantly limited [9-11].

Prior work has argued that the core issue is not that patients do not have the ability to understand test results but that the current design of patient portals inhibits effective result comprehension [12]. That is, many portals only present test results to patients in a table, which was originally formatted for clinician interpretation [11,13,14]. As a result, lay individuals, especially those with low health literacy and numeracy, have difficulty identifying meaningful information from their laboratory test results, such as how concerning the results are and what they should do to cope with them [15]. It is therefore imperative to ensure that patients not only have access to their laboratory test results but are also able to *understand* and *act upon* them.

To achieve this goal, the key challenge to address when communicating laboratory test results to patients is how to optimize the way the results are presented [10] and provide patients with the necessary information to comprehend each result [12,16]. However, research on these aspects is limited. Only a few studies have explored the perceptions of patients on viewing laboratory test results via portals [12,17,18] and what visual cues might be useful for aiding result comprehension [19-22]. However, little is known about how to design *patient*-*centered* technology support to promote the interpretation of test results on the part of the patients and, in turn, improve patient-provider communication [16].

Furthermore, individual patient characteristics (eg, age and sex) and medical contexts (eg, health issues and chronic conditions) are different from each other. Accordingly, the interpretation of a similar laboratory value may differ on the basis of the health condition of a patient. For example, the standard reference range of a laboratory test may not be applicable to older adults with chronic conditions [10]. Prior work has found that people often provided personal health information (eg, laboratory test results, age, medical history, and lifestyle) when posting questions on health forums to receive personalized recommendations from their peers [23,24]. From this perspective, providing information support tailored to the medical contexts of the patients could be very useful for them to determine whether results are worrisome and what might be appropriate for them to do.

In this study, we began our inquiry by asking the following research questions: (1) How to design patient-facing interfaces or tools to improve comprehension of laboratory test results for lay patients with average health literacy? (2) What system features are deemed useful (or not useful)? (3) What kinds of concerns or barriers do patients have regarding such patient-facing applications? To that end, we conducted a mixed methods, user-centered research focused on designing and evaluating an interactive prototype for communicating laboratory test results in a way that can support understanding and informed decision-making for everyone from different medical contexts and with different characteristics. More specifically, through user studies, we identified the information and technology needs of the patients related to viewing and interpreting laboratory test results. The user needs assessment informed the design of the system prototype. Finally, we conducted a pilot user evaluation with 8 patients to obtain feedback on individual design features and informational support in the system. Through this user-centered, multicomponent design exploration, we make the following contributions to the field of health informatics:

1. Design concepts for an interactive system to support patient-centered communication and comprehension of laboratory test results, as well as discussions between patients and health care providers.
2. Design implications for informative, trustworthy, and empathy-driven technology support in the context of communicating health data to lay patients.

### Related Work

#### Patient-Centered Communication of Laboratory Test Results

The communication of clinical information, such as laboratory test results, has historically taken place during face-to-face clinical encounters. However, previous literature has pointed out various drawbacks with regard to relying solely on in-person discussions of clinical information. For instance, patients often have difficulties contacting their physicians and, thus, are not able to receive timely explanations of their laboratory test results [25]. Even during clinical visits, various barriers may hinder effective communication between patients and their physicians and, consequently, the questions the patients have are sometimes left unanswered [26-28].

Advances in patient-facing technologies, such as patient portals, enable patients to directly access laboratory test results and other personal clinical data outside of the clinical environment. The benefits of increasing the access of the patients to their data are numerous, such as enhancing patient-centered medical care [6], improving patient engagement in decision-making [1,4,5], and empowering patients to play an active role in their health care management [29]. However, the use of patient portals to review laboratory test results among patients remains limited [30]. The reason for this is multifaceted. For instance, the current interfaces of patient portals mostly present laboratory test results to patients in a tabular format, similar to the format seen by clinicians, making it challenging for patients to make sense of them [11,13,14]. Furthermore, patients with limited health literacy and numeracy find it hard to understand complex health concepts and make meaningful use of their laboratory test results (eg, determining whether they should be concerned and take action immediately) [11,31]. These challenges highlight the importance of addressing the needs and preferences of the patients when designing technology support for communicating laboratory test results. As indicated by prior work, failing to involve patients in the design process of patient-facing applications might lead to issues with technology adoption and usability [32]. To this end, our research takes a user-centered approach to explore design concepts and considerations while taking into account the informational and technological needs of the patients.

#### Technology for Supporting Comprehension of Laboratory Test Results

Given the complex nature of clinical data, seminal research has attempted to design health information technologies to improve people’s use of clinical data. Hong et al [14] designed a system prototype to support patients, families, and health care providers in collaboratively reviewing radiology imaging data during in-person clinical visits [14]. Similarly, Arnold et al [33] developed a radiology patient portal interface to provide explanations of medical terms in lay language to help patients understand how to review radiology images. These studies have demonstrated novel techniques for supporting patient-centered communication of complex clinical data.

Despite the critical role of laboratory test results in diagnosing and screening for diseases, it remains largely unexplored how technology should be designed to support their comprehension outside of clinical settings, when the informational support that usually takes place during in-person patient-provider communication is absent. A notable exception is the study conducted by Nystrom et al [16], who designed and evaluated a new laboratory test result interface for patient portals, consisting of visual ranges of laboratory values and nontechnical descriptions of the tests. These features were deemed useful because they accounted for the needs of the patients [16]. However, one limitation of this study is that they did not address how to help patients understand the connections between their medical context and test results, and the necessary support and actions after receiving these test results. Our study bridges this important research gap.

## Methods

This study consists of multiple components: user studies, an iterative prototype design, and a pilot evaluation study. All studies were approved by the Pace University Institutional Review Board.

### User Studies and Prototype Design

To understand how to better support the interpretation of laboratory test results on the part of the patients through novel patient-facing technology (research question 1), we first conducted a web-based survey with 203 participants and a set of semistructured interviews with 13 patients in 2019. The user studies focused on the confusion and faced challenges of the patients pertaining to the interpretation of laboratory test results and on the informational and technological needs of the patients for better comprehension of test results. All interviews were audiotaped with the permission of the participants. Detailed information about the methodology of the user studies was reported in our previous publication [34].

The research team then used the results of the user studies to inform the design of a software prototype supporting patient-centered communication of laboratory test results. The prototype was designed in an iterative manner—after creating a design version, the researchers shared it with a small group of interview participants (n=3) for quick feedback and design improvements. This process lasted from January to June 2020.

### User Evaluation

Following the prototype design, we conducted a pilot evaluation study through which we obtained responses from patients regarding individual design features and the information presented in the prototype. This evaluation study helped us answer research questions 2 and 3. We recruited 8 participants who had recently used patient portals to review laboratory test results. The demographic information is summarized in Table 1. All evaluation sessions were conducted remotely via Zoom between July and August 2020. Each session lasted 60 to 90 minutes. The consent form and a short demographic questionnaire were sent to the participants before the scheduled session. During each session, we first informed the participants about the purpose of our study and confirmed their consent to take part in it and be audio-recorded. A weblink to the prototype was sent to them so that they could explore the prototype system during the study session.

**Table 1:** Characteristics of the participants in the user evaluation study.

Participant ID	Sex	Age (years)	Frequency of reviewing laboratory test results^a^	Health literacy^b^
P1	Male	26-49	2-5 times	4
P2	Male	18-25	2-5 times	3
P3	Male	50-64	Once	5
P4	Female	50-64	Did not remember	5
P5	Female	18-25	Once	4
P6	Female	26-49	2-5 times	5
P7	Female	26-49	6-10 times	4
P8	Male	26-49	Once	3

^a^The number of times a participant used a patient portal to review their laboratory test results over the previous 6 months.

^b^Health literacy was self-reported by the participants on a scale of 1 to 5 (*1* denoted low literacy and *5* denoted high literacy).

We started with a demonstration of the system prototype to explain the features of the system and design rationale for each feature, as well as how patients could leverage these features to interpret their results. The participants were then encouraged to use the prototype on their own to learn more about the system. During this process, they were asked to *think aloud* [35] by reporting their general perceptions of the system, anything that confused or surprised them, or anything that did not fully meet their expectations or needs. Since the goal of the evaluation was to obtain feedback on system features, we asked the participants to provide responses regarding whether each feature was useful rather than the nuanced design details (eg, color scheme and font size).

Once the system demonstration was completed, the researchers conducted a follow-up semistructured interview with each participant to further inquire about their experience and perceptions of using the system prototype. In particular, we sought to obtain user feedback regarding individual features, visual design, presented information, and usefulness of the system prototype. The session was concluded by administering a System Usability Scale (SUS) questionnaire—a 10-item attitude Likert scale for subjective assessments of system usability [36]. The purpose of administering the SUS questionnaire was 2-fold: (1) to assess the usability of the system prototype and (2) to collect baseline data to measure improvements for future prototype refinements. Each session was audiotaped, including verbal comments and questions from the participants about the prototype.

### Data Analysis

The audio recordings of the evaluations were transcribed verbatim, and the transcripts were imported into NVivo (version 12; QSR International) for qualitative analysis. Two researchers (ZZ and LK) followed an iterative, inductive coding method [37] to analyze the transcripts and met regularly to discuss and refine codes until no new codes emerged. In the second round of analysis, coded data were grouped under themes using affinity diagrams [38]. Themes and subthemes were discussed iteratively among the researchers until a consensus was reached.

## Results

In this section, we first present how the results of the user studies informed the design of a prototype application supporting patient-centered communication of laboratory test results. We then report the findings of the evaluation study.

### Prototype Design for Supporting the Comprehension of Laboratory Test Results

#### Summary of the Results of User Studies

In this section, we briefly summarize the principal findings of the user studies to contextualize our following descriptions of how the user studies informed the system design. The detailed results of the user studies were reported elsewhere [34].

##### Confusion in Reviewing Laboratory Test Results

We found that there were various sources of confusion for patients regarding their test results. For example, the test report used medical jargon excessively, which is not comprehensible for lay individuals. In addition, patients were confused about how to interpret their results. In particular, when abnormal results were received, patients could not determine how serious they were. Finally, patients found it challenging to make sense of the results and their implications for their overall health care, especially when the explanations of the physicians were lacking.

##### Information Needs

We found that patients needed different types of information to address their confusion, including both general and personalized information. More specifically, general information needs were related to the medical terminology, reference range, and diagnostic abilities of a specific test. In contrast, many participants emphasized the importance of receiving personalized information on the basis of their medical context. First, they desired to understand the implications and causes of such abnormal test results, as well as how serious they were and if immediate action was needed. Second, there was a demand for more information about treatment options, including medications and medical procedures available for treating the medical conditions indicated by the abnormal results. Finally, a few participants wished to be informed about what actions to take next.

##### Technology Needs

With respect to what kind of technology support could aid their comprehension, patients emphasized that technologies should be designed for patient interpretation. In particular, the system should be user-friendly and accessible for marginalized groups (eg, older adults) to minimize disparities in the use of health technology [39]. Other approaches that were deemed useful included (1) visualizing historical results, (2) using lay terms to communicate the nature of the results, (3) including a health encyclopedia to explain medical terms, and (4) leveraging artificial intelligence to provide more personalized medical information on the basis of the medical conditions of individual patients.

#### Design Goals

On the basis of the results of the user studies [34], we established a list of design goals.

##### Facilitating Result Comprehension Using Graphical Representations and Clear Takeaway Messages

Currently, patient portals present laboratory test results in 2 main ways: dichotomously (normal vs abnormal) or through numerical values. Even though the standard reference range for each test is usually provided, patients still have difficulties understanding the seriousness of their results and whether differences between a test result and the standard reference range are significant [10,34,40]. On the basis of the literature, providing clear, plain language indications [19] and graphical representations [22,41,42] makes laboratory test results easier to review and interpret. For example, visual aids can be used to illustrate whether a result is beyond a clinically worrisome threshold. Our prototype design experimented with these cues.

##### Enabling Annotation of Test Reports

Patients often have a variety of questions about the different aspects of laboratory test reports. We believe that it is critical to enable patients to identify what section of the results they wish to look into or discuss further with clinicians [14]. To that end, we sought to provide annotation tools to allow patients to highlight and annotate certain medical terms and content in the application.

##### Clarifying Medical Jargon Using Nontechnical Verbiage

Owing to gaps in the medical domain knowledge of laypersons, the participants cited difficulties understanding medical jargon as a major barrier to the effective interpretation of test results. The literature highlights the importance of using patient-friendly language to facilitate health information–seeking and understanding, as well as informed decision-making, on the part of the patients [43-45]. Therefore, in our design, we used concise, nontechnical verbiage to describe and explain medical concepts.

##### Providing Pertinent and Reliable Information Tailored to the Medical Contexts of Individual Patients

When patients attempted to make sense of their results, they often turned to the internet to seek further information [23]. However, information on the web is sometimes either too general or misleading. For example, standard reference ranges of laboratory tests are not applicable to some patients with chronic conditions [10]. Our user studies indicated that patients wanted not only general information (eg, reference range and medical terms) but also personalized information (eg, treatment options, prognosis, and what to do or ask next) situated within the medical context of the patient to make sense of the normality and indications of the results. As such, our prototype was designed to present reputable and relevant information resources to help patients make sense of their results [46].

#### Prototyping System Features

##### Procedure

We created a system prototype on the basis of our design goals. Because many adult patients are very likely familiar with the lipid profile (a group of laboratory blood tests on a patient to identify various levels of fat substances. Specific tests include total cholesterol, low-density cholesterol, and high-density cholesterol)—a commonly performed laboratory test among adults for many screening and diagnostic procedures—we chose to base our prototype design on this laboratory test. We used the Figma prototyping software (macOS version) to create the design. With high-fidelity animations and page transitions, this prototype enabled the user to explore how the application functions and learn each feature in an interactive manner. An HTML version of the prototype was generated for future use (eg, sent to users for feedback). In the subsequent section, we describe the main features of the prototype and how each one supports the review of the test reports.

##### Result Presentation

**Figure 1:**
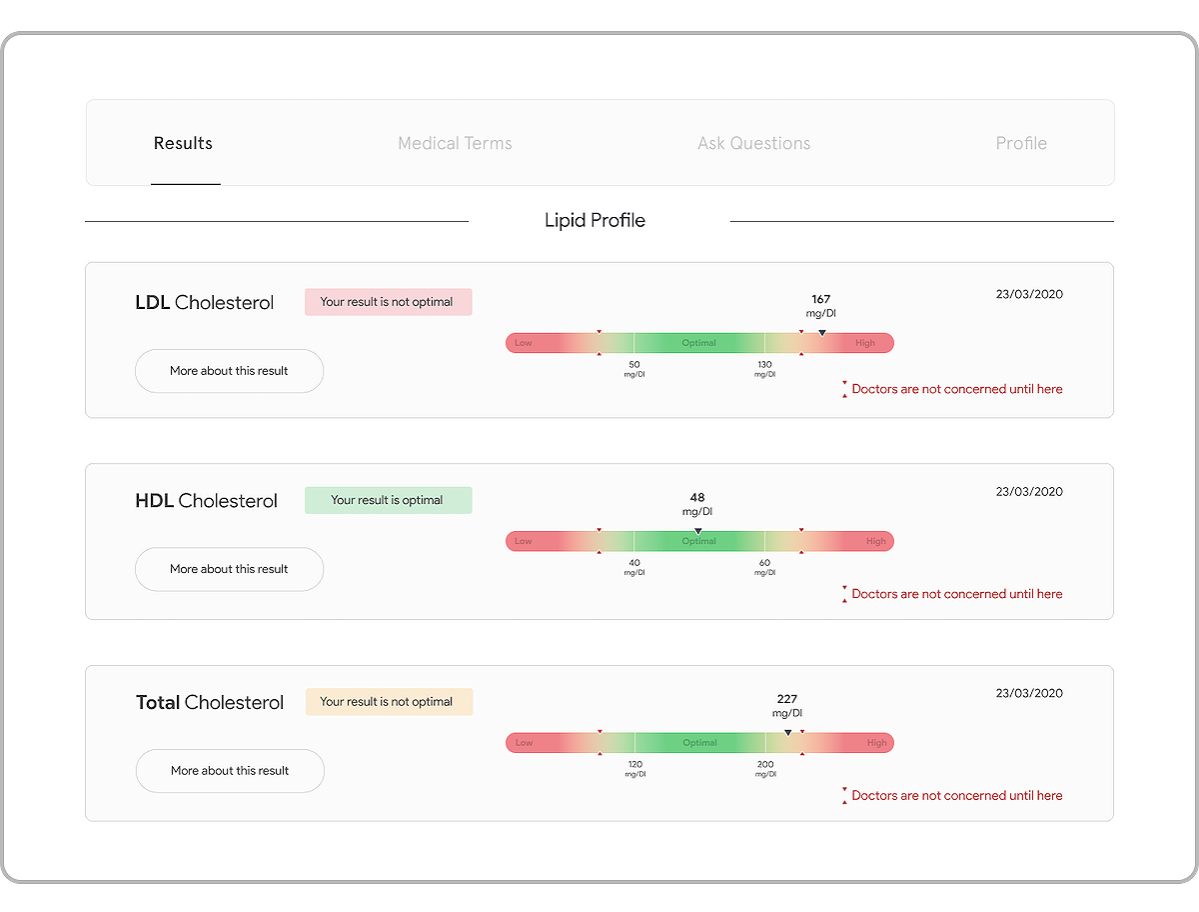
New result presentation interface. HDL: high-density lipoprotein; LDL: low-density lipoprotein.

We used graphical representations, meaningful plain language, and takeaway messages to construct a new presentation interface for laboratory test results. As shown in Figure 1, gradients of 3 colors (red, yellow, and green) were used in conjunction with words (eg, *high*, *low*, and *optimal*) and takeaway messages (eg, *your result is good*) to provide an intuitive view of the normality of each test value. To further enhance the patients’ understanding of the borderline values that were slightly outside of the normal range, we included a pair of red arrows and a side note (*Doctors are not concerned until here*) on each result chart to indicate at what point outside of the standard reference range the results become clinically concerning [21]. We believe that these visual aids and takeaway messages can help patients better understand the nature of their results. For example, the sample low-density cholesterol value is beyond the *clinically concerning* point, whereas the out-of-range total cholesterol value is still within the safe threshold. To further distinguish the urgencies, the takeaway message for low-density cholesterol is in red, whereas the total cholesterol takeaway message is in yellow.

**Figure 2:**
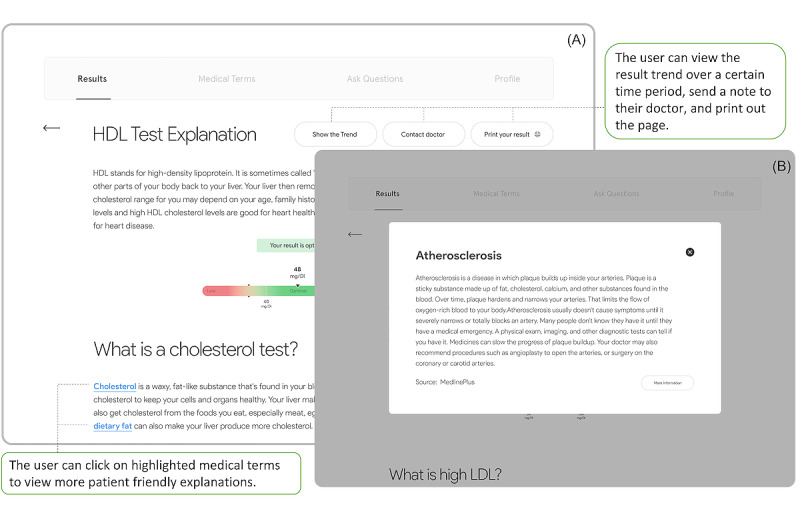
(A) Generic information about a laboratory test. (B) Explanations of a medical term. LDL: low-density lipoprotein.

In addition, the user was provided with an option to learn more about each laboratory test by clicking the *More about this test* button, which directed the user to a new page containing a brief definition, detailed explanations, and a visualization of historical laboratory values (Figure 2A). The brief definition explained what a specific laboratory test was testing. Users could also find more general information related to how to deal with abnormal results (eg, *What causes high cholesterol?*). The visualization of historical laboratory values presented the trend of test results over a customizable time period (eg, 6 months or a year). This visualization could help identify the level of variation between a new result and previous results.

##### Medical Terms

Professional medical terms in the text were highlighted in blue and made clickable for the patient to learn more (Figure 2A). When a linked term was clicked, a short and concise definition was displayed in a pop-up window (Figure 2B), where users could choose to view more detailed, patient-friendly explanations retrieved from MedlinePlus (a web-based health information resource and service for patients provided by the National Library of Medicine). The sources of these explanations were noted.

**Figure 3:**
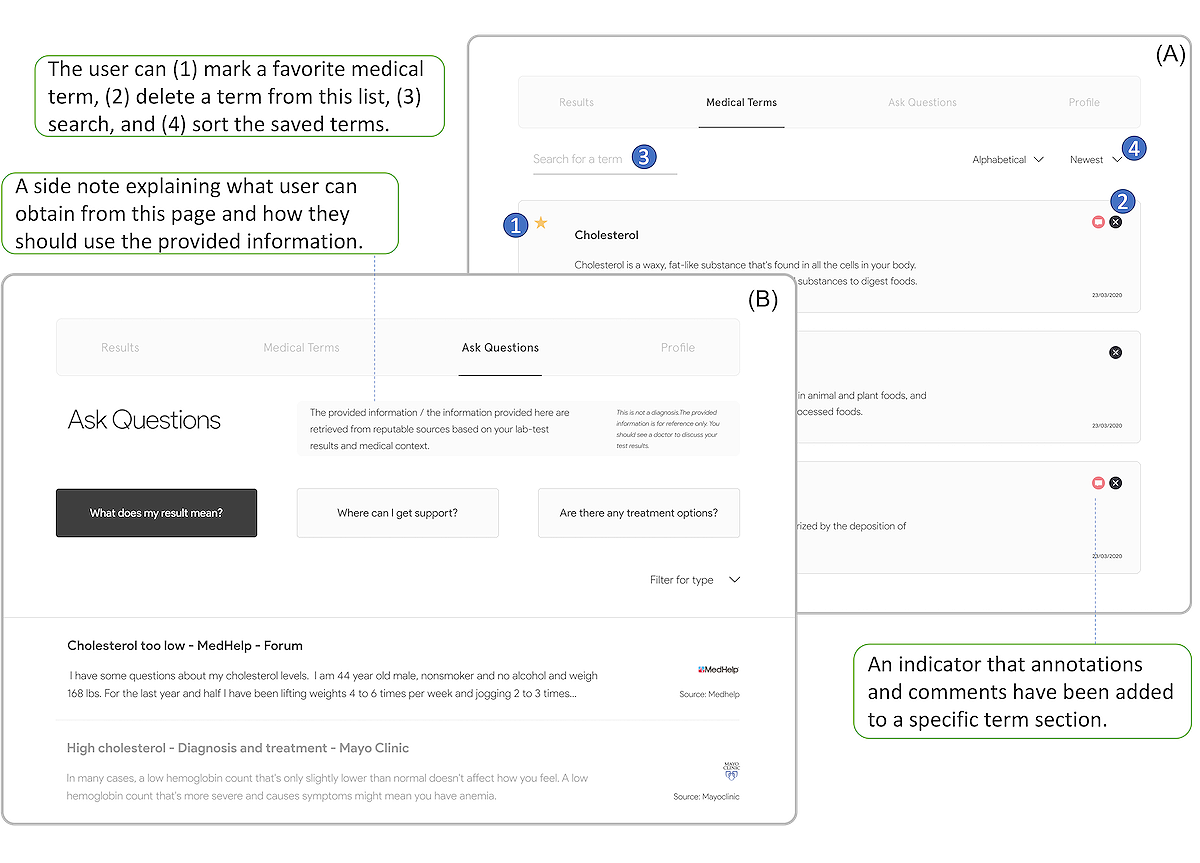
(A) Medical Terms page. (B) Ask Questions page.

In addition, every medical term the patient clicked was automatically saved to a separate page, called *Medical Terms* (Figure 3A), for future use. The user could favorite, annotate (eg, add a comment), or simply delete each saved term. All comments and annotations could be stored on the interface for later viewing by patients or clinicians (an indicator appeared if comments had been added to a specific term section). Favorited terms were always displayed at the top, followed by other saved terms. All terms added to this page could be easily searched or sorted.

##### Ask Questions

The *Ask Questions* section was designed to provide *personalized* information support tailored to the demographics, medical context, and laboratory test results of the patient. On the basis of the user study results, we decided to include 3 commonly asked questions in the interface: “What does my result mean?” “Where can I get support?” “Are there any treatment options?” (Figure 3B). Information related to these 3 questions could be retrieved in real time from reputable web sources, such as health care organizations (eg, Mayo Clinic), online health care forums (eg, MedHelp), and medical literature databases (eg, PubMed). The original source of the presented information was also provided, enabling patients to decide whether they wanted to trust and use that information. In light of medical ethics [47], users were advised that the information was for reference only and that the application was not designed as a diagnostic tool.

Since the primary goal of this study was to explore and evaluate design concepts and considerations rather than implementing a fully functional system, we decided to hardcode information on the basis of a hypothetical patient case. For example, we conducted a search on MedHelp using hypothetical laboratory values and patient age and sex and retrieved a post containing similar information. We then added that post to the “What does my result mean?” section and provided a link to the original post.

##### Other Features

The application provided an annotation tool on all pages, allowing patients to add comments and highlight texts. This tool was expected to facilitate reading of and reflection on the results and discussions between patients and physicians during in-person clinical visits.

The application also provided a straightforward onboarding process through which a patient could easily import their laboratory test report from the patient portal of the health care provider. The medical information (eg, chronic conditions) and characteristics (eg, age and sex) of the patient could also be imported and then stored in the *Profile* page.

### Pilot System Prototype Evaluation

In this section, we describe the pilot evaluation sessions with 8 patients and report their responses to individual design features and the information presented in the prototype.

#### Overall Perception

The participants uniformly acknowledged the significant improvement of the prototype over the current patient portal design and expressed excitement about using the new application to review and interpret their results:

It looks extremely robust and I am basing that on my comparisons of what I have now, which is nowhere near, doesn’t come anywhere near this functionality. So it’s a vast improvement over what I have now.

P3

The average SUS score was 95 (out of 100), signifying that the prototype was perceived as very user-friendly. In the subsequent section, we describe the detailed feedback on each of the main features of the system.

#### Result Presentation

All participants had a positive response to the new presentation interface of the test results. One primary improvement was the use of visual cues in combination with takeaway messages to intuitively communicate the nature of the results. As the participants stated:

I think the colors are really helpful and that having that indicator on the side correspond with the colors saying, “your whatever is good” and then you have “low” or “high” corresponds with the color. It’s all very easy to read. I really like the whole package, everything is very clean.

P7

I really like the “doctors are not concerned until here” thing, because sometimes my numbers are low. It’s not extremely low, but it’s still low, and I’m not sure if I should be concerned or not.

P2

The participants also appreciated the ability to learn more about each test:

You have the very obvious button to get more information that you might need. I think for that use, it’s perfect for what you have on there. Not too much, not too little. I think it’s pretty good.

P8

When asked about what aspects of the new result presentation interface could be further improved, the feedback was mostly focused on the wordings. For example, 2 participants mentioned that some words in the takeaway messages (eg, *not optimal*) could trigger unnecessary anxiety. They suggested that the communication of abnormal laboratory test results to patients should take their emotions into consideration:

You don’t want to panic anyone. You don’t want anyone to read anything that they’re going to immediately have to be on the phone with their doctor, right? [. . .] Maybe just keeping that in mind when you have the results page set up. [. . .] Having some sort of caveat that these blood results are just results they’re not diagnose. [. . .] You need to be empathetic when giving bad results.

P6

#### Medical Terms

All participants agreed that automatically highlighting medical terms in the text and providing detailed explanations with patient-friendly language was very useful, and this convenience of getting to know medical terms could improve the experience of reviewing test results:

It’s probably one of the most useful things. Because when I am interpreting my personal lab results, I’d be back and forth going to a search engine to look up and find out what it is. That would save me the trouble of doing that if I could just click on it.

P3

With regard to automatically saving every clicked or viewed medical term to a dedicated page (*Medical Terms*), we received diverse feedback. Most participants (n=6) stated that they found it very useful for future use, but some concerns were raised. For example, one participant mentioned that she might not be interested in using and reviewing the *Medical Terms* page:

I do think it is useful but I don’t know if it’s totally necessary like the Results section is. I could see me not really using the Medical Terms section as much as the Results or the Ask Questions section.

P7

Another participant pointed out that users may not be aware that the clicked medical term was automatically saved to a new page for future use. As such, they suggested redesigning this user flow to increase awareness of this potentially useful feature.

#### Ask Questions

All participants considered the *Ask Questions* section one of the most useful features because it provided pertinent information tailored to the medical context of individual patients:

I think it is actually potentially one of the coolest parts of the whole thing for every different condition that a person could have and for all the different things a person could have on their chart. If it all comes up with really good, concise information for that person to take further, I could see this being incredibly useful.

P8

When asked if including only 3 prescribed questions was sufficient, the participants had positive responses:

I would say that those three questions cover what would effectively be my general concern.

P1

However, they also suggested adding “what to ask during a clinical visit” to this page:

It might be cool to have a section or information about what you should ask your doctor.

P7

Even though we specified the sources of the provided information, some participants (n=3) still had concerns regarding information trustworthiness because each person has their own trusted information sources. For example, the information collected from well-known health organizations was deemed more reliable than the information from peer-to-peer online forums:

If you took me to a peer to peer forum, I wouldn’t trust it since they are not always the most reliable thing and there’s a lot of misinformation there. I think if you would like to use such things, you might want to always have a caveat with something like “please be aware of peer to peer responses.” You get my point? [. . .] You should link more to credible information sources, such as JAMA or Lancet.

P4

#### Annotation

The perceived usefulness of the annotation tool varied among the participants. Some (n=4) acknowledged that it was useful to take notes for future use, remarking that:

I’m going to look for the things in here that I feel like are the most important and I would definitely annotate those so that I can scroll quickly back through it if I needed a refresher.

P1

However, others (n=4) stated that they very likely would not use this tool, as one participant explained:

Personally, I think its usefulness is marginal. I probably wouldn’t use it. I’m probably going to come here [the application] and I’m going to read what I want to read and pick up what I want to pick up. That’s not saying that someone else might not want to highlight some things, maybe they would. [. . .] But I doubt seriously that personally I would use it. I mean, it’s kind of cool, but I don’t think I’d really miss it if it wasn’t there.

P3

Another participant echoed this statement and further explained that she was a “paper person,” so she was not comfortable using the digital annotation tool:

Personally, I don’t really think that I would use it. I would print this out and highlight stuff to show or read when I see my doctor.

P2

During follow-up discussions on how to make the annotation tools more useful, half the participants (including one who previously had a positive attitude in this regard) suggested consolidating all the annotations in one page for easy reading, management, and retrieval:

It would be really cool to have a section sort of like the “Medical Terms” where you can save questions to ask your doctor. So you can just go to your doctor’s office for the follow-up and pull up the app and ask the questions right from the app.

P7

Another participant shared a similar idea and expressed the desire to be able to share all the notes and questions with his primary physician before the clinical visit:

We all know that we only have 15 minutes with our doctors, and they prefer we email them most of the time now. If you could export the notes, and your questions about what you saw here into one document, and then get them to my doctor through a messenger, that would be great.

P4

#### General Feedback

Interestingly, the participants did not express any privacy concerns about the use of their personal health information to generate personalized information support:

Any realm of health care is so highly regulated. I understand that there are breaches, but I don’t personally have that concern. So I feel like if people have already used patient portals then they’re not necessarily going to have those concerns and the ones that are super concerned aren’t going to use any sort of patient portal.

P1

In fact, they stated that they understood why their personal health data were needed, and they were willing to provide more information to receive tailored support.

Regarding the aspects of the application that could be improved, the participants provided some interesting suggestions. For example, one participant raised that people with different levels of health literacy may need different types of information and, as such, the system should be designed to first assess their health knowledge and then present information tailored to their literacy level:

So, if I’m new to the medical condition, it is going to be different than if it’s something that I’ve had for a while and I understand some of the medical terminologies, right? So it may be useful to have a variety of information sources and suggestions for the user with different health literacy backgrounds. You can have very basic things for a new patient but for someone that has had the medical condition for a while, you are going to want maybe some academic articles with most recent research results or that kind of thing.

P6

In addition, some participants recommended adding explanations to the presented information to avoid potential confusion or misunderstandings:

The one thing that you might want to have a little caveat somewhere, [stating] that those ranges are created based on what is normal for a certain percentage of a population so if you’re outside of range doesn’t necessarily mean that you will show signs and symptoms. [. . .] You can have a little note as to where these ranges come from so when people look at their result and see high or low, where they are on the spectrum, they are having an idea of where those come from and if the normal ranges apply to him or her.

P6

Finally, it was deemed useful to provide both a web and mobile version of the system to expand its use scenarios, for example, it is easier to use mobile version during in-person clinical visits, whereas many users preferred a web-based version at home:

Having a mobile application with you while you’re at your doctor visit might help to more quickly review the results and get your doctor on board.

P1

## Discussion

### Principal Findings

In this study, we designed and evaluated an interactive system prototype to support patients in making sense of their laboratory test results. The system design was informed by user studies, the details of which were reported elsewhere [34]. The system prototype consisted of several novel, interactive features: (1) using graphical representations and clear takeaway messages to convey the nature of the results, (2) enabling users to annotate laboratory test reports, (3) clarifying medical jargon using nontechnical verbiage and allowing users to interact with the medical terms (eg, saving, favoriting, or sorting), and (4) providing pertinent and reliable information tailored to the medical contexts of individual patients. Through a pilot user evaluation, potential users uniformly acknowledged the significant improvement of the system over current patient portals in communicating laboratory test results to patients. They noted that the new system could facilitate their interpretation of laboratory test results by promoting self-education regarding different aspects of laboratory test reports.

In addition, we identified two other use cases in which the new system could play a significant role. One was helping patients be better prepared for clinical consultations. Although it varies by health care setting, there is evidence suggesting that clinicians are spending less time with each patient and are often unable to fully address their questions [48]. Even more concerning is that some clinicians frequently interrupt patients, making it difficult for them to have their questions answered during clinical encounters [49]. Our application can better prepare patients for clinical consultations so that they can use their time with physicians effectively. For example, both general and personalized information provided in the application could help the patient research different aspects of the laboratory report and, in turn, contemplate and devise questions related to the results before the consultation.

The other use case regarded facilitating patient-provider discussions during clinical visits. For example, the application allows patients to annotate and document comments or questions while viewing the results. These annotations can be used later during clinical visits to facilitate patient-provider discussions. Although not mentioned by the participants, we envision that these annotation tools can also be used by patients to take notes during doctor visits. Patients can then use the notes to support memory recall of the information discussed during the consultation. Our future work will iteratively design and evaluate interactive features, such as annotation tools, that can further enhance in situ review of laboratory test data with clinicians.

### Design Implications

#### Informative Technology Support

It is common to see patients feeling helpless when they receive laboratory test results because current patient portals provide limited support for them to assimilate the information and make informed decisions. Our user studies highlighted the importance of providing additional information that patients could read more about to begin conducting their own research [34]. For example, even though 2.5 mU/L in the first trimester and 3.0 mU/L in the second and third trimesters are considered the standard reference ranges for the thyroid-stimulating hormone during pregnancy, it was pointed out that these cut-offs are too low and may lead to overtreatment [50]. It might be useful to provide this information to pregnant patients to raise their awareness.

Furthermore, the participants appreciated the ability to receive information tailored to their medical context so that they could learn what the results meant, what they could do next, and where to obtain support. A key example was individualizing the standard reference range—given that a typical standard reference range for laboratory test results is developed on the basis of a large, healthy population, it may not be applicable to certain populations, such as pregnant women, older adults, or people with comorbidities [10]. In this case, it is useful to individualize the frame of reference by allowing custom reference ranges. Given that many known or unknown factors could affect what reference ranges might be desired for a patient, this may be more appropriately done by their health care provider, in which case they should be granted access to the system as well.

#### Promoting Trust

One major feature of our application is the provision of additional information tailored to the medical context of the patient. Despite its perceived usefulness, some participants expressed concerns about the credibility and trustworthiness of the information provided. In this prototype, we provided the original source of the information to alleviate this issue. In-depth analyses of trusted information sources revealed that some people preferred information provided by well-known health care organizations, whereas a few others wished to read scientific literature published by reputable medical journals. From this perspective, it is necessary to tailor the delivery of additional information to the individual differences of the patients. One way to accomplish this is by allowing patients to customize their trusted and preferred information sources within the application to individualize information delivery. In addition, it would be helpful to provide a mechanism for patients to rate the usefulness and trustworthiness of each information source, and the ratings can be used to create a curated list of reputable information sources.

Future development of personalized support in this context is expected to rely on advanced machine learning techniques, which can take patient characteristics, medical contexts, and laboratory values as inputs to find and retrieve relevant and up-to-date medical evidence. The literature on the perceived trustworthiness of machine learning and artificial intelligence technologies has suggested presenting a variety of system-related information to the users to help them better understand how the recommendations of the system are generated and then determine whether it is appropriate to trust them [51]. For example, Lee and See [52] suggested presenting system reliability (eg, how reliable and accurate the system is) and logic and reasoning (eg, how the system operates), as well as the types of information that the system leverages (or excludes) to generate recommendations. In future work, aligning with these suggestions, we will examine whether providing more appropriate explanations for how the system generates medical advice could be of any help in promoting acceptance on the part of the patients and fostering trust in the application.

#### Empathy-Driven Design

In the medical field, the types of clinical data that should be shared with patients and how the data should be communicated have been the subject of debate among informatics researchers [53]. Some clinicians are concerned about providing patients with direct access to clinical data, such as laboratory test results, before clinical consultations [54]. The primary reason is that clinicians worry that patients, especially those who receive abnormal test results, are vulnerable to anxiety and frustration when reviewing the results without the presence of clinicians [12]. In contrast, patients are increasingly interested in having direct access to their test results, regardless of their normality [4,18]. The key, then, is how to communicate test results and their potential implications to patients while accounting for their emotional needs. Prior work has suggested that patient-facing applications that communicate sensitive information (eg, abnormal laboratory test results) should be designed with *empathy*. However, such needs have been largely unfulfilled by current patient-facing systems, even though the empathy-driven design has been gaining momentum for many years [55].

In our prototype design, we took into account this design consideration. For example, we followed suggestions by prior work [21] to provide visual aids to indicate at what point outside of the standard range the results become clinically worrisome with the aim of lowering patient distress if they receive slightly out-of-range test values. We also provided a list of web-based sources in the *Ask Questions* section, where patients could seek and receive emotional support. In future work, we will examine additional features and strategies that can mitigate emotional stress. For example, as one of the participants suggested, it might be useful to add a simple caveat in plain language to address the concerns of the users (eg, “this standard range is only applicable to 80% of people, many factors, such as chronic health conditions and characteristics, could impact what might be the appropriate frame of reference for you”). We will also work closely with communication specialists and medical professionals to synthesize best practices and strategies for communicating abnormal results to patients and then incorporate them into the system refinement.

### Conclusions and Limitations

In this paper, we described a multicomponent study focused on the design of a patient-centered system prototype to help patients interpret highly professional laboratory test results. Through user studies, we identified patient informational and technological needs specific to this domain, which were then used to inform a set of design considerations and concepts. After iterative prototyping, an initial evaluation study was conducted to obtain feedback from 8 patients, who uniformly had positive responses and acknowledged the significant improvement over existing patient portals in supporting comprehension of laboratory test results on the part of the patients. Finally, on the basis of our findings, we discussed design implications for communicating personal clinical data to lay individuals in a more informative, trustworthy, and empathetic manner.

A few limitations of this study should be noted. First, we only conducted an initial evaluation study with a small sample size (n=8). However, they all had extensive experience with laboratory tests and had reviewed laboratory test results via patient portals. All of them acknowledged the significant improvement of our application design over the current patient portals. We will use the feedback received to refine the system prototype. Second, in the pilot evaluation, our focus was mainly on the responses of the patients to each design feature or concept with respect to whether or not the design or system feature was useful. As such, we primarily collected subjective assessment data. We did not evaluate the knowledge gain of the patients in using our system or the extent to which the system could help patients make sense of each result and inform their decision-making and subsequent actions. These aspects will be assessed in future user evaluation sessions. Third, we used a hypothetical scenario with only one type of laboratory test (lipid profile). This limitation could affect the generalizability of the results of our study. As we continue to refine the prototype and address its shortcomings, we plan to explore how these design concepts and considerations play out in different medical scenarios, as well as whether they are able to support the interpretation of different types of laboratory tests and can be used by different patient populations. Finally, because this was a user-centered design exploration study to investigate design considerations for supporting patient comprehension of laboratory test results, we did not fully implement the system. This is a common practice in user-centered design research [56]. For example, to prototype the feature of presenting relevant information tailored to the context of the patient, we manually searched information on the web based on the hypothetical patient case, collectively determined which information source to use, and then hardcoded the information into the application. In future work, we will implement the system and integrate it with existing patient portals once the system design is finalized.

## Abbreviations

SUS: System Usability Scale
